# Novel insights into the N 6-methyladenosine modification on circRNA in cancer

**DOI:** 10.3389/fonc.2025.1554888

**Published:** 2025-05-19

**Authors:** Qingling Xu, Yi Jia, Ying Liu, Bing Wu, Jianxun Wang, Xiang Ao, Wei Ding

**Affiliations:** ^1^ Department of Comprehensive Internal Medicine, The Affiliated Hospital of Qingdao University, Qingdao, Shandong, China; ^2^ School of Basic Medicine, Qingdao University, Qingdao, Shandong, China; ^3^ Institute for Translational Medicine, The Affiliated Hospital of Qingdao University, Qingdao Medical College, Qingdao University, Qingdao, Shandong, China

**Keywords:** circRNA, N6-methyladenosine, cancer, m6A modification, drug resistance

## Abstract

Circular RNAs (circRNAs) are a class of non-coding RNAs (ncRNAs) generated through the reverse splicing of mRNA precursors (pre-mRNAs). They possess a unique loop structure and exhibit remarkable stability. CircRNAs have emerged as promising biomarkers for cancer, with specific circRNAs playing crucial roles in cancer drug discovery, treatment, and resistance mechanisms. N6 methyl adenosine (m6A) represents the most prevalent RNA modification in eukaryotes. In 2017, researchers identified that m6A modifications also occur in circRNAs, displaying unique characteristics. m6A-modified circRNAs undergo reversible regulation mediated by enzymes involved in m6A modification pathways. These modified circRNAs interact with m6A-binding proteins, thereby influencing processes such as alternative splicing, translation and degradation. Some circRNAs enhance their metabolism or facilitate nuclear export to the cytoplasm by interacting with enzymes involved in m6A regulation. The study of m6A-modified circRNAs has gained great attention in circRNA research due to their association with various diseases. This review summarizes the functional mechanisms of circRNAs regulated by m6A modifications and their implications in cancer occurrence and therapy, with a primary focus on the genesis, regulatory mechanisms, and functional roles of m6A-modified circRNAs in the biology of diverse types of cancers. Additionally, we explore the potential application of m6A-modified circRNAs in clinical cancer treatment.

## Graphical Abstract

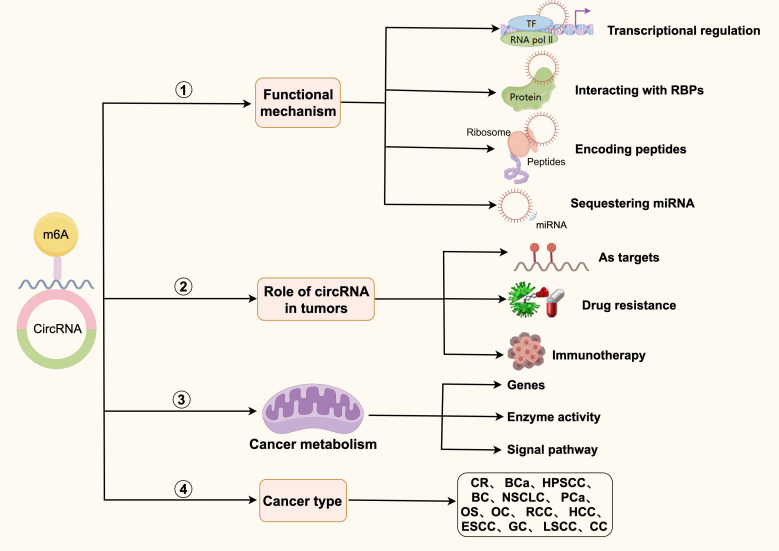

## 1 Introduction

Cancer remains a leading cause of mortality worldwide (1–3). Its development is characterized by intricate alterations in multiple processes and the dysregulation of numerous factors (4). A key contributor to this complexity is the disruption of cellular metabolism and signaling pathways that govern cell growth, leading to uncontrolled alterations in energy production and metabolic requirements that promote cell proliferation (5). Despite significant progress in therapeutic approaches such as chemotherapy, radiotherapy, surgery, endocrine therapy, and other approaches (6, 7), the efficacy of cancer treatment remains unsatisfactory, particularly for patients with advanced stages of the disease. This limitation can be attributed to tumor cells’ ability to evade death when exposed to therapeutic stress, thereby developing resistance against treatments. Additionally, the effectiveness of different anti-cancer medications may also be compromised due to changes in the transportation, metabolism, and interactions with drug targets. Another important factor is that tumor cells themselves can gain survival advantages through mechanisms that prevent cell death, repair DNA damage, activate autophagy, modify the tumor microenvironment (TME), induce epithelial-mesenchymal transition (EMT), and other processes. Although substantial advancements have been made, further elucidation of the pathogenesis of cancer is essential to identify more precise treatment strategies. Circular RNAs (circRNAs) exhibit strong associations with the growth, programmed cell death, and invasion of various cancer cells highlighting their potential as novel therapeutic targets or biomarkers (4).

CircRNA, a unique type of non-coding RNA (ncRNA) generated through specialized splicing processes, exhibits distinct characteristics such as its stable circular structure. It plays a vital role in the biological processes underlying various diseases, particularly cancer. Recently, there has been growing interest in elucidating the involvement of circRNA in the treatment of cancer drug resistance. Multiple studies have identified circRNAs as potential targets for therapy and biomarkers for cancer. In diverse disease contexts, circRNA has been shown to regulate key cellular processes, including autophagy (8), apoptosis (9), cell cycle (10), and proliferation (11). Consequently, it holds promising prospects for therapeutic applications. Research on the relationship between circRNA and cancer can be broadly categorized into two aspects: one aims to utilize differential expression levels of circRNA in cancer tissues as potential diagnostic markers for cancer (12), while the other investigates the regulatory roles of circRNA in cancer development and progression (13).

With the increasing depth of circRNA research, it has been demonstrated that N6-methyladenosine (m6A) modification can occur in circRNAs, which is one of the most prevalent RNA modifications in eukaryotic cells. This reversible modification plays a critical regulatory role in various aspects of RNA metabolism, including transcription, processing, splicing, and translation (14–17). The regulatory function of m6A is primarily controlled by three similar factors referred to as ‘writers,’ ‘erasers,’ and ‘readers’. These factors are responsible for the reversible methylation of m6A-modified RNA and identification of the modified sites (18–21). In 2017, Cell Research published the first evidence demonstrating m6A modification in circRNA and its potential to enhance circRNA translation. Subsequently, Cell Reports confirmed that m6A modification is widely present in circRNAs (22). Further analysis conducted on human embryonic stem cells (hESCs) validated the presence of m6A modification in both circRNAs and their corresponding linear RNAs (23). CircRNAs with m6A modifications have been implicated in various diseases including cancer (24), immune system disorders, neurodegenerative diseases (25), and cardiovascular diseases (26).

This comprehensive review systematically summarizes recent advancements in elucidating the mechanisms by which m6A modifications contribute to cancer pathogenesis, circRNA-related therapies, and their clinical applications.

## 2 m6A modification of circRNA

### 2.1 Overview of circRNA

As a distinct class of endogenous ncRNAs, circRNAs form a covalently closed loop structure via a reverse splicing mechanism (27). In contrast to other RNA species such as mRNAs, microRNAs (miRNAs), and long noncoding RNAs (lncRNAs), circRNAs lack a 5′-terminal cap structure and a 3′-terminal poly(A) tail, enabling them to form a specialized loop structure. This unique structural feature renders circRNA is less susceptible to degradation by ribonuclease R (RNase R), thereby enhancing their stability (28).

**Figure 1:**
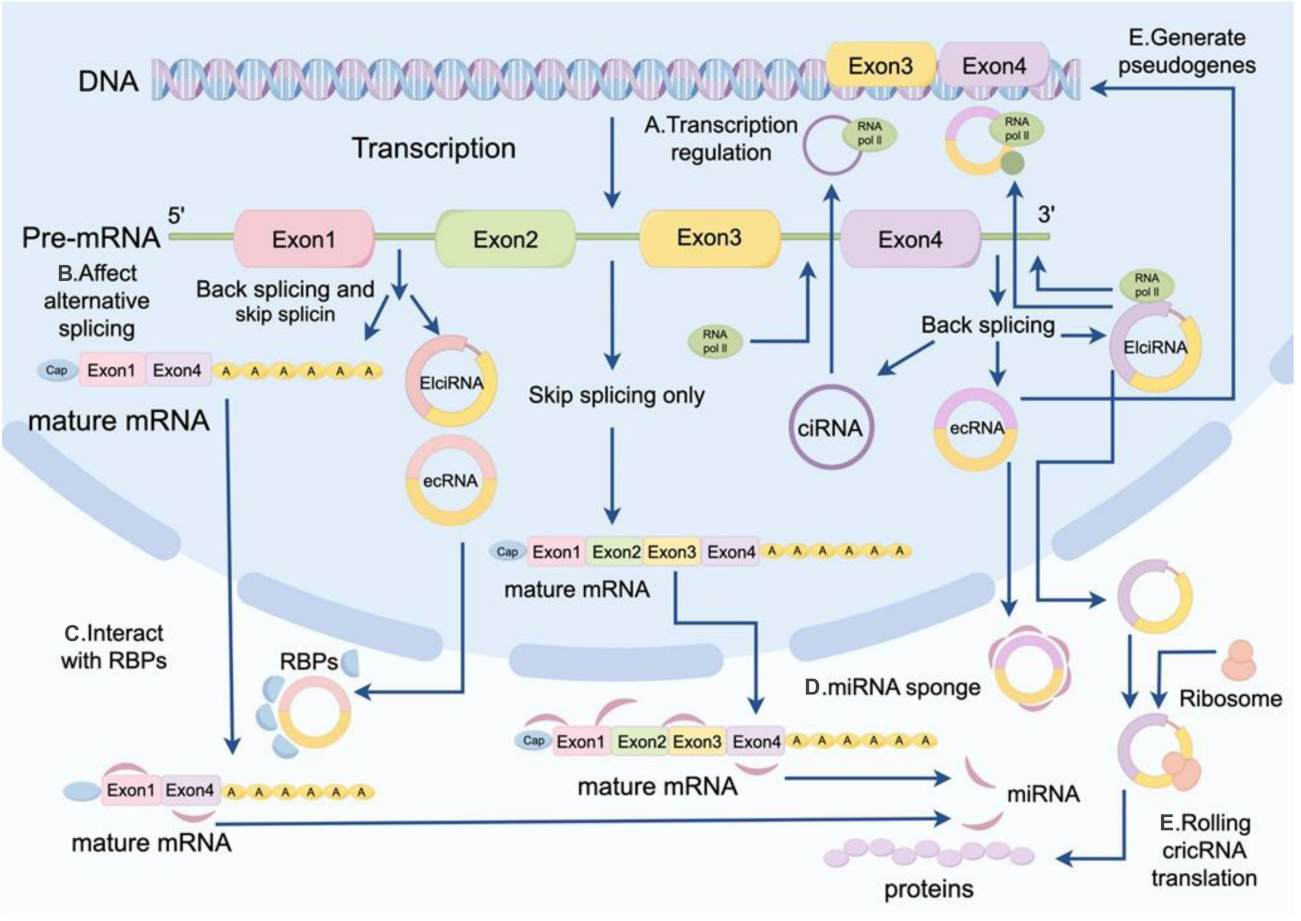
Functional mechanisms of circRNAs. Biological functions of circRNAs include transcriptional regulation, splicing regulation, binding to RBP to exist as a protein scaffold, miRNA sponging, and protein translational regulation.

CircRNAs play crucial roles in transcriptional regulation, splicing regulation, and translational regulation (**Figure 1**). As transcriptional regulators, circRNAs regulate the transcription process of genes by interacting with RNA polymerase II (Pol II) or other transcription factors (29). Additionally, circRNAs can hybridize with DNA to form R-loop structures, thereby influencing gene transcription. For instance, circSEP3 in Arabidopsis thaliana induces transcription pauses and generates alternatively spliced mRNAs by forming R-loops with parental DNA sites (29). CircRNAs also participate in splicing regulation by affecting splice site selection, facilitating exon skipping, and acting as sponges for splicing factors (29). Furthermore, circRNAs act as miRNA sponges by binding to miRNAs, preventing their interaction with target mRNAs and indirectly regulating the expression of downstream target genes (30–32). For example, in Parkinson’s disease, the upregulated expression of circSNCA and circPANK1 promotes dopaminergic neurons degeneration by inhibiting miR-7, which increases the expression of α-synuclein (33). Moreover, some circRNAs contain internal ribosome entry sites (IRES), which can directly recruit ribosomes and initiate translation to produce biologically functional proteins. circFGFR1 drives translation through its IRES to produce proteins with dominant negative regulatory functions (33). Additionally, circRNAs can influence the translation efficiency of mRNAs by interacting with translation-associated factors (29).

In summary, circRNAs exhibit multifaceted functions in the regulation of gene expression, and their mechanisms of action are complex, diverse, and dependent on their interactions with other molecules. As research progresses, the significance of circRNAs in biology and medicine is expected to become increasingly prominent.

### 2.2 m6A modification

The m6A modification represents one of the most prevalent and abundant types of RNA modification in eukaryotes (34). It plays a crucial role in the entire RNA life cycle, encompassing transcriptional regulation, maturation, translational regulation, degradation, and stability maintenance of mRNAs (34). m6A methylation is a dynamic and reversible process mediated by three key regulators involved in m6A modification: the enzyme responsible for adding methyl groups to mRNAs (referred to as the “m6A writer”), the enzyme responsible for removing methyl from mRNA (referred to as the “m6A remover”), and the protein responsible for recognizing m6A-modified sites (referred to as the “m6A reader”) (35). The regulatory enzymes and recognition proteins are summarized in **Table 1**.

**Table 1:** M6A regulatory protein details.

Type	Factor	Mode of action	Reference
Writers	METTL3	The main methyltransferase.	(36)
	METTL14	Form heterodimer with METTL3.	(37)
	WTAP	Stable METTL3-METTL14 complex	(38)
	RBM15/15B	Directs METTL3-METTL14 heterodimer to specific sites.	(39)
	VIRMA	Auxiliary METTL3-METTL14 complex positioning.	(40)
	ZC3H13	Combine with WTAP.	(41)
	METTL16	Catalyzes m6A modification.	(42)
Erasers	FTO	Removes m6A modification.	(43)
	ALKBH5	Removes m6A modification.	(44)
	ALKBH3	Removes m6A modification.	(45)
Readers	YTHDF1	Promotes mRNA translation.	(46)
	YTHDF2	Promotes mRNA degradation.	(47)
	YTHDF3	Cooperate with YTHDF1 or YTHDF2.	(48)
	YTHDC1	Promotes mRNA splicing and export.	(49)
	YTHDC2	Improve the translation efficiency of target mRNA.	(50)
	IGF2BP1	Promotes the stability and translation of mRNA.	(49)
	HNRNPC/G	Regulate the abundance and splicing of Mrna.	(51, 52)

m6A modification plays a regulatory role in biological processes through the dynamic interplay of “writers", “erasers” and “readers". m6A methyltransferases, known as “m6A writers", promote mRNA modification through the addition of m6A. The m6A methyltransferases, known as “m6A writers", promote mRNA modification by adding m6A. METTL3, the core catalytic enzyme for m6A modification, is responsible for methylating the N6 position of adenosine in RNA. It forms a heterodimer with METTL14 to enhance its catalytic activity (53). Additionally, WTAP, a key auxiliary protein for m6A modification, facilitates the localization of the METTL3/METTL14 complex to nuclear speckles and enhances its catalytic activity (54). The assistance of the auxiliary ligand protein RBM15/15B is also required to help the METTL3-METTL14 heterodimer to be spliced correctly, to ensure that the methyltransferase complex is correctly localized in the nucleus while maintaining its stability, and to recruit specific RNA substrates (39). In addition to the canonical m6A modifications, an independent RNA methyltransferase called METTL16 has been identified, which catalyzes m6A modifications on the 3’UTR and U6 mininucleotide RNAs of mRNAs (42). m6A modifications are introduced by the methyltransferase, which can be subsequently removed by the m6A demethylase (34). m6A demethylases include mainly Fat mass and obesity-associated protein (FTO) and ALKB homolog 5 (ALKBH5). FTO was first identified in 2011 as the first demethylating enzyme capable of reversing m6A modifications *in vivo*, significantly stimulating interest in the study of m6A modifications (43). Subsequently, ALKBH5 was also identified as another key enzyme involved in m6A removal. ALKBH5 plays an important role in a variety of cancer-related biological processes and represents one of the hotspots of research in this field (44). In addition to m6A writers and erasers, m6A modification involves a key class of enzymes, the m6A methyl-recognizing enzymes, often referred to as “m6A readers”. These readers primarily consist of the YTH N6-methyladenosine RNA-binding protein (YTHDF) family. In the cytoplasm, the YTHDF family consists of YTHDF1, YTHDF2, and YTHDF3, each of which plays a different role. Specifically, YTHDF1 promotes the translation of mRNAs, whereas YTHDF2 mainly contributes to the degradation of mRNAs; comparatively, YTHDF3 is also involved in promoting mRNA translation (44, 46, 48). Additionally, the insulin-like growth factor-2 mRNA-binding protein (IGF2BP) family also plays a key role as another important m6A readers. In contrast to the YTH structural domain family proteins, which promote mRNA degradation upon binding to m6A-modified RNA molecules, the IGF2BP family proteins can stabilize mRNAs (49).

In summary, m6A modification, as a pivotal epigenetic modification, plays a crucial role in the regulation of gene expression and various biological processes through its dynamic and reversible regulatory mechanism. Future studies are expected to further elucidate its specific functions under diverse physiological and pathological conditions and investigate its potential as a therapeutic target for diseases.

### 2.3 Regulation of circRNA by m6A modifications

**Figure 2:**
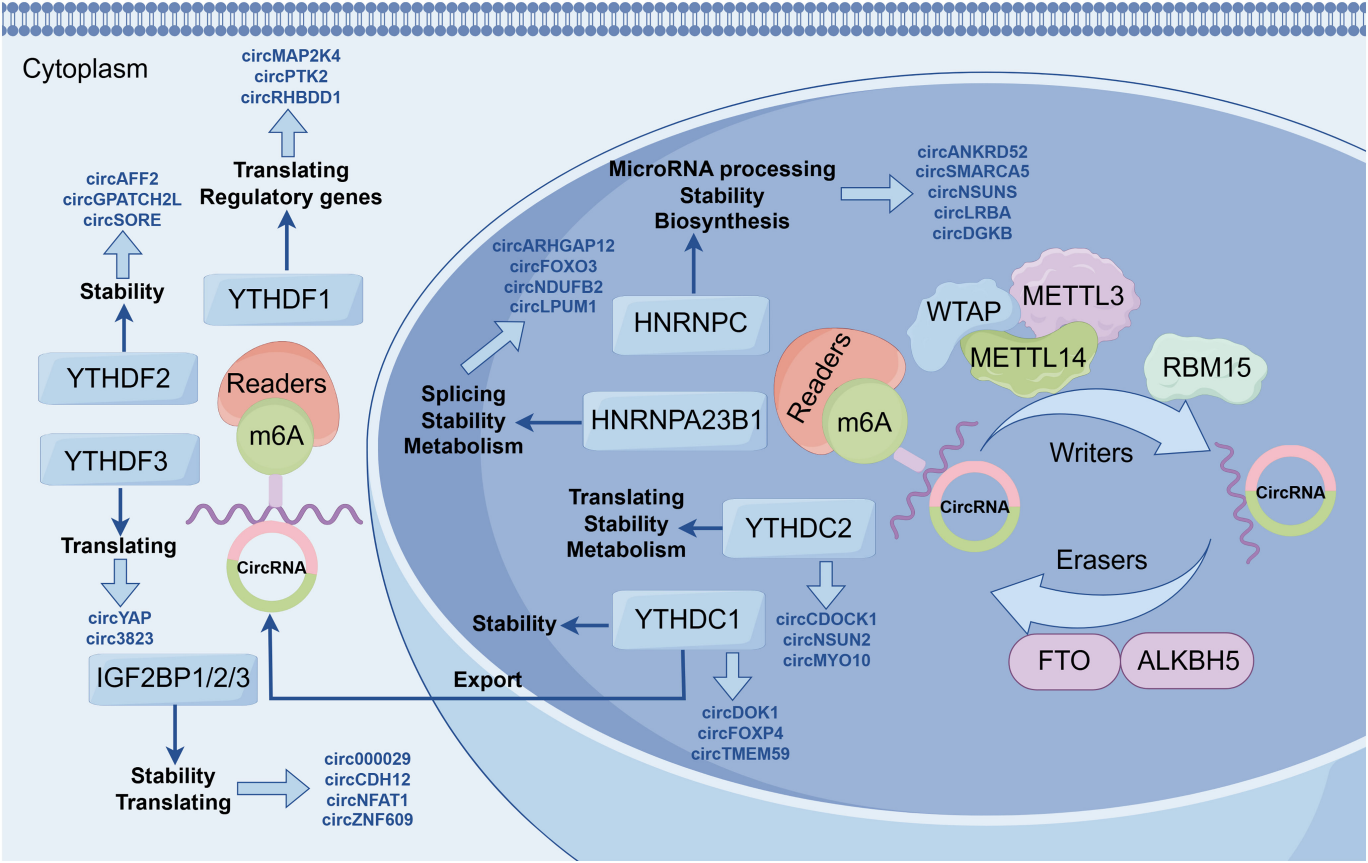
Regulatory mechanisms of m6A-modified circRNAs. These mechanisms include transcriptional regulation, splicing regulation, and translational regulation.

A recent study demonstrated that circRNAs are direct targets of m6A modification (55). In recent years, the understanding of the functional mechanisms of m6A-modified circRNAs in diseases has been gradually deepening. Here, we summarized that m6A-modified circRNAs play crucial roles in transcriptional regulation, splicing regulation, and translational regulation (**Figure 2**).

The m6A modification represents one of the most abundant RNA modifications in eukaryotes and can significantly influence circRNA stability and metabolic processes. Studies have demonstrated that m6A modifications regulate the intracellular localization and stability of circRNAs by interacting with “reader” proteins such as YTH family proteins (56). For instance, m6A modifications can facilitate the nuclear-to-cytoplasmic transport of circRNAs, thereby enhancing their potential to act as miRNA sponges or translational templates (57). Moreover, m6A modifications play a critical role in circRNA biosynthesis. By interacting with splicing factors, m6A modifications can modulate exon selection and splicing efficiency, thereby influencing circRNA formation (58). In some cases, m6A modifications can promote the jumping of specific exons, thereby affecting circRNA production (57). m6A-modified circRNAs can regulate the translation process through multiple mechanisms. On the one hand, m6A modification can enhance the stability of circRNAs so that they stay in the cytoplasm for a longer period of time, thereby increasing their chances of serving as miRNA sponges or translation templates. On the other hand, m6A modification can promote circRNA translation. For example, has been shown that m6A modification enhances the activity of the internal ribosome entry site (IRES) on circRNAs, thereby initiating the translation process. Furthermore, m6A modifications can also regulate circRNA function during translation by modulating their interactions with RNA-binding proteins (57).

In summary, m6A-modified circRNAs play pivotal roles in transcriptional regulation, splicing regulation, and translational regulation. The realization of their functions depends on the precise modulation of circRNA metabolic processes mediated by m6A modifications. These findings provide valuable insights into the role of circRNAs in gene expression regulation and identify potential targets for the diagnosis and treatment of associated diseases.

## 3 The role of circRNA in tumorigenesis and therapy

### 3.1 circRNAs as promising biomarkers for cancer diagnosis and prognosis

In recent years, substantial advancements have been achieved in the investigation of circRNAs as potential biomarkers for cancer. CircRNAs possess unique structural features, such as a covalently closed loop structure, a long half-life, and cell-type specificity, which allow them to show great potential in cancer diagnosis and therapeutic monitoring (59).

CircRNAs are highly expressed in the plasma and blood of patients with various cancers and can serve as potential biomarkers for early disease detection, particularly for cancers that are typically diagnosed at late stages, such as pancreatic ductal adenocarcinoma and glioblastoma (59). Additionally, alpha-fetoprotein (AFP) and alpha-fetoprotein agglutination reaction have been utilized for diagnosing and predicting the prognosis of hepatocellular carcinoma (HCC) (60). By analyzing and normalizing microarrays data from HCC tissues and adjacent non-tumor tissues, differential expression patterns of multiple circRNAs were identified in HCC tissues (61). Notably, elevated ciRS-7 levels were found to correlate with microvascular invasion and AFP levels in HCC patients (62). These findings suggest that circRNAs hold promise as diagnostic and prognostic markers for HCC. Despite their cell-type specificity, ideal tumor biomarkers should exhibit exclusive expression in tumors. Moreover, smaller and more heterogeneous tumors may not produce sufficient circRNAs to be detected by conventional assays (e.g., PCR), necessitating the development of more sensitive but potentially costly assays, which remains a technological challenge for researchers to address.

### 3.2 circRNAs and cancer chemotherapy resistance

The role of circRNAs in cancer chemoresistance is gradually gaining attention. Studies have shown that circRNAs are involved in the process of chemoresistance in cancer through multiple mechanisms and are expected to be a new target for drug resistance research and treatment (63–65).

There is a wide variety of chemotherapy drugs, and doctors can choose the most effective drug based on the type of cancer and the specific needs of the patient. However, cancer cells may gradually develop resistance to chemotherapeutic drugs, leading to a decrease in treatment efficacy. However, circRNAs play a key role in resistance to cancer chemotherapy and targeted therapy drugs. Studies have shown that circ0004674 develops resistance to chemotherapy in osteosarcoma cells and tissues, thereby affecting their prognosis. This resistance may be achieved through modulation of apoptosis-related pathways (66). Further studies on drug resistance showed that circPAN3 acts as a mediator of drug resistance in acute myeloid leukemia through the miR-153-5p/miR-183-5p-XIAP axis (67). This mechanism provides important new insights into the role of circRNAs in mediating drug resistance in AML. In addition, circAKT3 showed high level expression in cisplatin (DDP)-resistant cancer cells and tissues. Similarly, circ0081143 promotes DDP resistance by regulating the miR-646/CDK6 pathway. Knockdown experiments targeting these molecules were able to inhibit tumor formation while significantly increasing the sensitivity of cancer cells to DDP (63). These findings provide new insights into addressing drug resistance during cancer treatment.

The existence of drug resistance poses a major challenge to cancer treatment. CircRNA’s mechanism of action in cancer chemotherapy resistance is becoming clearer, and its research as a marker of drug resistance and a therapeutic target is being deepened, providing new ideas and directions for cancer treatment. Future studies will further reveal the specific mechanism of circRNA in drug resistance and explore its clinical application value.

### 3.3 The potential of circRNA in tumor immunotherapy

In recent years, the prospect of circRNA application in tumor immunotherapy has gradually attracted attention. circRNAs have the advantages of high stability, low immunogenicity and tissue-specific expression, which make them show great potential in tumor immunotherapy (68).

circRNAs can regulate the tumor immune microenvironment and affect the expression of immune checkpoints through multiple mechanisms. In non-small cell lung cancer (NSCLC), circFGFR1 acts as a competitive endogenous RNA for miR-381-3p and regulates the expression of CXCR4. Inhibition of CXCR4 enhances the sensitivity of NSCLC cells to PD-1 immunotherapy, suggesting that circFGFR1 may promote resistance to anti-PD-1 therapy (69). CircRNAs can serve as vaccine vectors that encode tumor antigens to elicit immune responses. Small circRNA vaccines delivered via lipid nanoparticles are able to express antigens consistently for more than a week *in vivo*, triggering robust T cell responses. In a mouse model, the small circRNA vaccine significantly suppressed a variety of poorly immunogenic tumors, including melanoma resistant to immune checkpoint blockade (70), when combined with immune checkpoint inhibitors. Thus, further exploration of the involvement of circRNAs in cancer immune responses and tumor immunotherapy will greatly contribute to the discovery of more convenient ways to treat cancer. In addition, it was found that lysogenic poxvirus-mediated antitumor effects could be regulated through the circRNA-103598/miR-23a-3p/interleukin-6 axis. In addition, many tumor-expressed circRNAs can be secreted into the bloodstream via exosomes with high stability and enrichment (69). Thus, circRNAs can be used as tumor markers in liquid biopsies for tumor detection and prediction of immunotherapy efficacy.

CircRNAs show potential for multiple applications in tumor immunotherapy, including modulation of immune checkpoints, as vaccine carriers, and as liquid biopsy markers. With deeper research and technological advances, circRNAs are expected to provide new strategies and methods for tumor immunotherapy, bringing more therapeutic options to patients.

## 4 m6A modifications mediate circRNA metabolism in cancer

### 4.1 Metabolism-related gene expression

m6A modification indirectly affects the expression of metabolism-related genes by regulating circRNA stability. It has been shown that m6A modification can regulate the adsorption ability of circRNAs to miRNAs, thereby deregulating the inhibitory effect of miRNAs on metabolism-related genes. tIGAR (TP53-induced regulator of glycolysis and apoptosis) plays a key role in regulating metabolic reprogramming in cancer cells, and changes in its expression level directly affect the proliferative capacity of cells (34). This mechanism provides new perspectives for understanding the molecular basis of cancer and offers potential targets for developing new cancer therapeutic strategies.

### 4.2 Metabolic enzyme activity

The m6A modification is one of the most abundant RNA modifications in eukaryotes, which is widely involved in gene expression regulation and plays an important role in metabolic reprogramming in cancer. Recent studies have shown that m6A modification promotes cancer development by regulating circRNA stability, subcellular localization and function, which in turn affects metabolic enzyme activities.

m6A modification indirectly affects metabolic enzyme expression by regulating circRNA stability. It has been shown that m6A modification can regulate the adsorption ability of circRNAs to miRNAs, thereby deregulating the inhibitory effect of miRNAs on metabolic enzyme genes (e.g., TIGAR) (71). m6A plays a key role in regulating metabolic reprogramming in cancer cells, and changes in its expression level directly affect the proliferative capacity of cells (71). m6A modifications not only affect the expression of metabolic enzymes, but may also directly or indirectly affect the activity of metabolic enzymes. For example, it was found that m6A modification affects the balance of glycolysis and the pentose phosphate pathway (PPP) by regulating the expression of TIGAR (71). In addition, m6A modifications may also promote metabolic reprogramming in cancer cells by regulating the expression of other metabolism-related genes and affecting metabolic enzyme activities. m6A modifications are aberrantly expressed in cancer and are closely associated with the activities of a variety of metabolic enzymes. For example, in hepatocellular carcinoma, high expression of METTL3 was positively correlated with G6PD expression, and patients with low expression of METTL3 and high expression of G6PD had a relatively better prognosis (71). This suggests that m6A modification may be a potential target for cancer therapy by regulating the activity of metabolic enzymes.

The m6A modification indirectly affects the expression and activity of metabolic enzymes by regulating circRNA stability, which in turn regulates metabolic reprogramming and proliferation of cancer cells. This mechanism provides a new perspective for understanding the metabolic regulation of cancer and offers potential targets for the development of new cancer therapeutic strategies.

### 4.3 Metabolic signaling pathway

In pancreatic cancer, m6A-modified circRNAs play an important role by regulating metabolism-related signaling pathways. A study found that METTL3-mediated m6A modification can significantly affect circCEACAM5 expression, which in turn promotes pancreatic cancer progression through activation of the DKC1 signaling pathway (72). Although this study did not directly explore how DKC1 affects metabolic signaling pathways, considering the critical role of DKC1 in cell proliferation and metabolism, it is hypothesized that it may promote metabolic reprogramming of pancreatic cancer cells by regulating intracellular metabolic enzyme activities or metabolic signaling pathways (e.g., PI3K/AKT, mTOR, etc.). This finding provides a new perspective for understanding the role of m6A modifications in the regulation of cancer metabolism and offers potential targets for the development of new cancer therapeutic strategies.

## 5 Interaction of m6A modifications with circRNAs in cancer development and therapy

**Figure 3:**
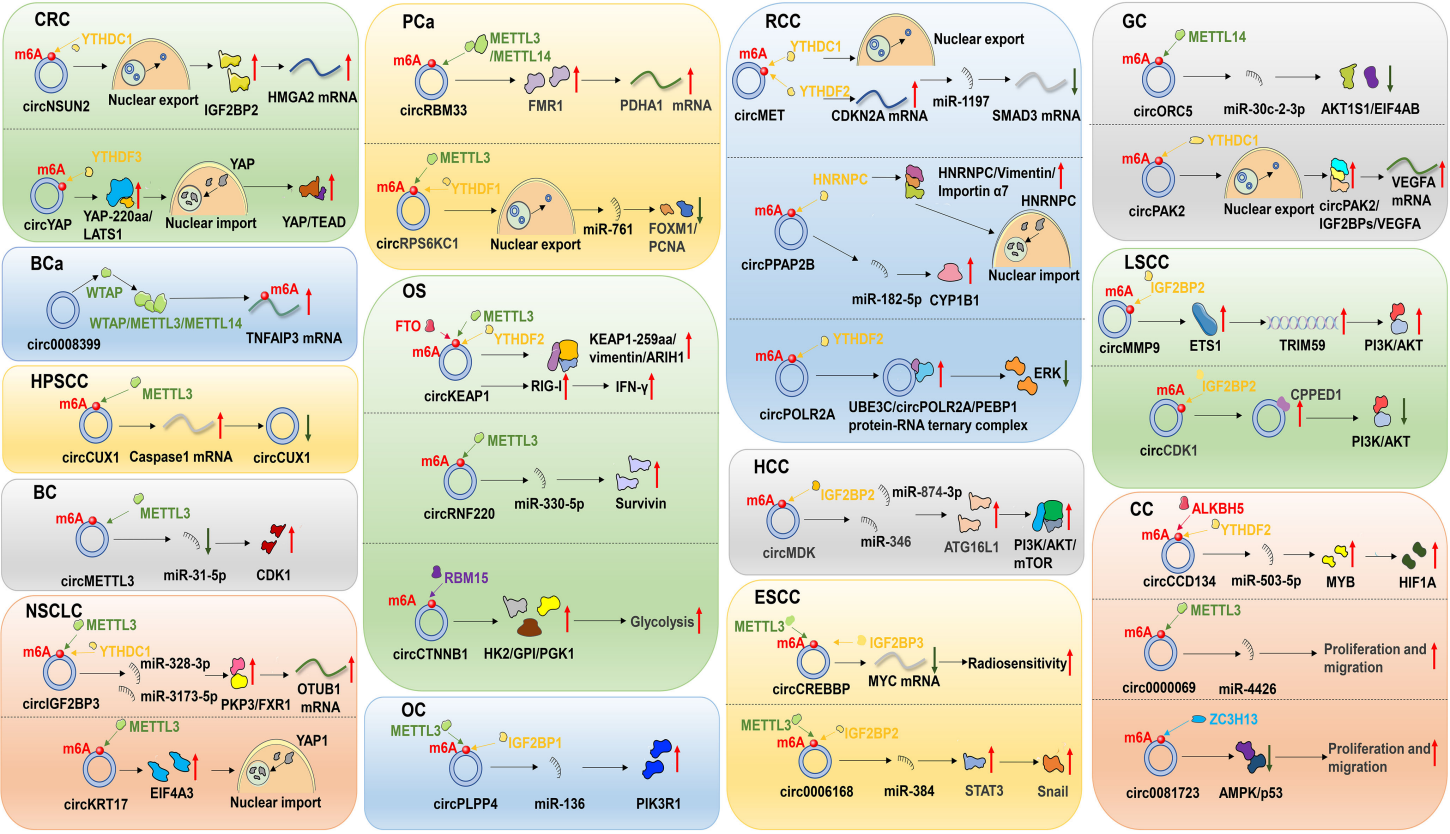
Regulatory mechanisms of m6A-modified circRNAs in the treatment of malignant tumors. These malignancies include colorectal cancer (CRC), bladder cancer (BCa), hypopharyngeal squamous cell carcinoma (HPSCC), breast cancer (BC), non-small cell lung cancer (NSCLC), prostate cancer (PCa), osteosarcoma (OS), ovarian cancer (OC), renal cell carcinoma (RCC), hepatocellular carcinoma (HCC), esophageal squamous cell carcinoma (ESCC), gastric cancer (GC), laryngeal squamous cell carcinoma (LSCC) and cervical cancer (CC).

With the deepening understanding of circRNA and m6A modifications, it has become increasingly evident that both circRNAs and m6A modifications play pivotal roles in cancer detection and treatment. Moreover, m6A modifications may influence the biological functions of circRNAs and contribute to their resistance to cancer therapies. This article offers a concise summary of recent studies on the regulatory mechanisms and functional roles of m6A-modified circRNAs in malignant tumor treatment (**Figure 3**, **Table 2**).

**Table 2:** m6A modified circRNA in various cancers.

Cancer type	circRNA	m6A modified enzyme	Mechanism	Reference
CRC	circNSUN2	YTHDC1	Facilitates its transport from the nucleus to the cytoplasm.	(73)
	circYAP	YTHDF3	Activates the YAP signaling pathway by blocking YAP phosphorylation through competitive binding to LATS1.	(74)
BCa	circ0008399	WTAP, METTL3, METTL14	Interaction with WTAP promotes the assembly of the WTAP/METTL3/METTL14 m6A methyltransferase complex.	(75)
HPSCC	circCUX1	METTL3	METTL3 promoted m6A methylation of circCUX1, thereby stabilizing its expression.	(76)
BC	circMETTL3	METTL3	Its expression was upregulated through the circMETTL3/miR-31-5p/CDK1 pathway.	(77)
NSCLC	circIGF2BP3	METTL3, YTHDC1	YTHDC1-dependent mechanism promotes its cyclization.	(78)
	circKRT17	METTL3	Nuclear localization of YAP1 was facilitated by recruitment of EIF4A3.	(79)
PCa	circRBM33	METTL3, METTL14	Regulation of PDHA1 mRNA stability by interaction with FMR1 protein.	(80)
	circRPS6KC1	METTL3, YTHDF1	Regulation of prostate cancer cell senescence through the FOXM1/PCNA axis.	(81)
OS	circKEAP1	METTL3, METTL14, YTHDF2	Regulates its stability by interacting with the m6A modifiers METTL3, FTO and YTHDF1/2.	(82)
	circRNF220	METTL3	Upregulates survivin expression by acting as a sponge for miR-330-5p.	(83)
	circCTNNB1	RBM15	Promotes m6A modification through RBM15 interaction to drive the glycolytic process.	(84)
OC	circPLPP4	METTL3, IGF2BP1	Mediation of cisplatin (CDDP) resistance in OC through the circPLPP4/miR-136/PIK3R1 axis.	(85)
RCC	circMET	YTHDC1, YTHDF2	Dependent m6A modification promotes its cytoplasmic translocation, thereby enhancing the decay of CDKN2A mRNA.	(86)
	circPPAP2B	HNRNPC	By interacting with HNRNPC to promote HNRNPC nuclear translocation.	(87)
	circPOLR2A	YTHDF2	By regulating UBE3C-mediated ubiquitination and degradation of PEBP1 protein and further activating the ERK pathway during cRCC progression and metastasis.	(88)
HCC	circMDK	IGF2BP2	Stimulation of the PI3K/AKT/mTOR signaling pathway through the miR-346/874-3p-ATG16L1 axis.	(89)
ESCC	circCREBBP	METTL3, IGF2BP3	Enhancement of ESCC radiosensitivity by reducing MYC mRNA stability through interaction with IGF2BP3.	(90)
	circ0006168	METTL3, IGF2BP2	The expression of circ0006168 in cells was increased in an IGF2BP2-dependent manner.	(91)
GC	circORC5	METTL14	Inhibition of GC progression by regulating the miR-30c-2-3p/AKT1S1 axis.	(92)
	circPAK2	YTHDC1	Interacts with IGF2BPs to form the circPAK2/IGF2BPs/VEGFA complex to stabilize VEGFA mRNA.	(93)
LSCC	circMMP9	IGF2BP2	Recruitment of ETS1 stimulates TRIM59 transcription, which in turn activates the PI3K/AKT signaling pathway.	(94)
	circCDK1	IGF2BP2	Activation of the PI3K-AKT signaling pathway via EIF4A3-circCDK1-IGF2BP2-CPPED1.	(95)
CC	circCCDC134	ALKBH5, YTHDF2	Fine-tuning by ALKBH5-mediated m6A modification enhances its stability in a YTHDF2-dependent manner.	(96)
	circ0000069	METTL3	Specific binding to sponge miR-4426 promotes CC cell proliferation and migration.	(97)
	circ0081723	ZC3H13	Promotes CC progression by regulating the AMPK/p53 pathway.	(98)

### 5.1 Colorectal cancer

In colorectal cancer (CRC), m6A-modified circNSUN2 has been shown to promote tumor liver metastasis. Research indicates that circNSUN2 expression is upregulated in the tissues and sera of patients with CRC liver metastases and correlates with poor prognosis. m6A-modified circNSUN2 facilitates nuclear-to-cytoplasmic transport by binding to the m6A recognition protein YTHDC1. In the cytoplasm, circNSUN2 interacts with the IGF2BP2 protein to form the circNSUN2/HMGA2 mRNA/IGF2BP2 complex, which enhances HMGA2 mRNA stability and facilitates CRC metastasis (73). Additionally, circRNAs can modulate the activity of m6A-modified enzyme through various mechanisms. For example, circYAP encodes a truncated YAP protein isoform (YAP-220aa) via m6A modification in CRC. This truncated isoform competitively binds to LATS1, blocking YAP phosphorylation and activating the YAP signaling pathway, thereby promoting tumor invasion and liver metastasis. Moreover, circ-YAP expression is transcriptionally regulated by YAP, forming a positive feedback loop that further drives CRC progression (74). The interplay between m6A modification and circRNA offers novel therapeutic targets for CRC treatment. In summary, the interaction between m6A modifications and circRNAs in CRC development and therapy holds significant biological implications and clinical application value. Future studies will further reveal its complex regulatory mechanism and explore its potential applications in CRC therapy.

Although some progress has been made in understanding the mechanisms of m6A modification and circRNA function in CRC, many questions remain to be further investigated. For instance, the regulatory mechanism of m6A modification on circRNAs may vary across different cancer types, necessitating additional experimental validation. Moreover, the roles of circRNAs and m6A modification in tumor immunotherapy warrant deeper exploration.

### 5.2 Bladder cancer

In bladder cancer (BCa) tissues and cell lines, eukaryotic translation initiation factor 4A3 (EIF4A3) promotes the upregulation of circ0008399 expression and inhibits apoptosis in BCa cells. Mechanistically, Wei et al. demonstrated that circ0008399 interacted with WTAP and facilitates the assembly of the WTAP/METTL3/METTL14 m6A methyltransferase complex, a finding that suggests targeting this axis may hold potential therapeutic value (75). Collectively, these findings provide potential therapeutic targets for circRNA-mediated m6A modification in BCa.

### 5.3 Hypopharyngeal squamous cell carcinoma

Hypopharyngeal squamous cell carcinoma (HPSCC) is a common malignancy in otorhinolaryngology head and neck surgery, with squamous cell carcinoma comprising over 90% of head and neck tumors (99). In radiotherapy-resistant HPSCC patients, circCUX1 exhibits upregulation, and a subsequent study demonstrated that METTL3 promotes m6A methylation of circCUX1, thereby stabilizing its expression (76). This finding underscores the potential of targeting circCUX1 modification by m6A as a therapeutic strategy to overcome radiotherapy resistance in HPSCC patients.

Currently, only limited progress has been made in elucidating the mechanisms of action of m6A modification and circRNAs in HPSCC, and further research is needed to explore the potential of m6A modification and circRNAs in the early diagnosis and personalized treatment of HPSCC.

### 5.4 Breast cancer

Breast cancer (BC) is the most commonly diagnosed malignancy in women and the leading cause of cancer-related deaths worldwide (100). CircMETTL3, a METTL3-derived cyclic RNA, has garnered significant attention in BC research due to its biological functions and potential mechanisms. In BC, circMETTL3 expression is markedly upregulated, promoting cell proliferation, migration, and invasion. m6A modification of circMETTL3 regulates its expression via the circMETTL3/miR-31-5p/CDK1 pathway, thereby driving BC progression (77). Additionally, METTL3, the host gene of circMETTL3, may regulate circMETTL3 expression in an m6A-dependent manner but does not affect METTL3 expression itself (77). This finding establishes a novel connection between circRNAs and their corresponding host genes, highlighting the potential therapeutic strategy of targeting circMETTlL3 for BC treatment.

### 5.5 Non-small cell lung cancer

Lung cancer is the leading cause of cancer deaths globally. Non-small cell lung cancer (NSCLC), which includes lung adenocarcinoma (LUAD) and lung squamous carcinoma (LUSC), represents the most prevalent form of lung malignancy (101). In NSCLC, overexpression of circIGF2BP3 suppresses T-cell activity, thereby impairing the immune response against tumor cells. However, METTL3 promotes m6A modification of circIGF2BP3, facilitating its cyclization via a YTHDC1-dependent mechanism (78). CircRNA microarray analysis revealed upregulation of circKRT17 and METTL3 in ositinib-resistant LUAD cells, and knockdown of circKRT17 and METTL3 enhanced the sensitivity of LUAD cells to ositinib. Mechanistically, METTL3 stabilizes circKRT17 by enhancing m6A modification, which promotes nuclear localization of YAP1 through recruitment of EIF4A3 (79). These findings offer novel insights into potential therapeutic strategies for ositinib-resistant LUAD patients.

### 5.6 Prostate cancer

Prostate cancer (PCa) is the most common non-skin malignancy among men globally. It is estimated that approximately 1.6 million men are diagnosed with PCa annually worldwide, and about 366,000 (102) die from the disease each year. In PCa, m6A-modified circRNAs may play an important role in tumor progression. Studies have demonstrated that circRBM33 expression is significantly higher in PCa cells compared to normal cells and tissues. CircRBM33 interacts with FMR1 protein via m6A modification to form a binary complex, which regulates PDHA1 mRNA stability and provides energetic support for the proliferation and metastasis of PCa cells (80). Additionally, m6A-modified circRPS6KC1 regulates PCa cell senescence through the FOXM1/PCNA axis, highlighting the importance of m6A modification in tumor cell senescence (81). The interaction between m6A modification and circRNAs in PCa development and therapy represents a complex yet promising research area. Future studies will further elucidate the specific mechanisms underlying this interaction and provide novel strategies for PCa diagnosis and treatment.

### 5.7 Osteosarcoma

Osteosarcoma (OS) is a malignant tumor originating from bone marrow plasma cells and predominantly occurs in the middle-aged and elderly population. In recent years, m6A modification-mediated circRNAs in OS have been extensively studied. In OS, the expression of circKEAP1 is regulated by m6A modification. It has been demonstrated that circKEAP1 interacts with the m6A modifiers METTL3, FTO, and YTHDF2, and the methylation status of its m6A modification site (A565G) regulates circKEAP1 stability (82). Additionally, METTL3-mediated upregulation of circRNF220 enhances survivin expression by acting as a sponge for miR-330-5p, thereby promoting OS progression (83). yang et al. revealed that circCTNNB1 promotes m6A modification through interactions with RBM15, driving glycolytic processes and activating OS progression (84). Collectively, m6A modifications and circRNAs exhibit complex interactions in OS development and treatment, influencing the biological behavior of OS by regulating each other’s expression and function. These findings provide novel insights into precision diagnosis and drug development for OS.

### 5.8 Ovarian cancer

Ovarian cancer (OC) is a highly aggressive malignancy that is often diagnosed at an advanced stage. Although this cancer initially responds well to platinum-based chemotherapy, the majority of patients experience recurrence following initial surgery and chemotherapy (103), highlighting the urgent need for new therapeutic strategies. The m6A-induced circPLPP4/miR-136/PIK3R1 axis mediates cisplatin (CDDP) resistance in OC, suggesting that circPLPP4 may serve as a promising therapeutic target for CDDP-resistant OC (85). Further investigation into the interactions between m6A modifications and circRNAs, as well as their regulatory mechanisms in OC, is expected to identify novel biomarkers and therapeutic targets, thereby providing innovative strategies for the diagnosis and treatment of OC.

### 5.9 Renal cell carcinoma

Renal cell carcinoma (RCC) is a malignant tumor originating from the epithelium of renal tubules and accounts for 80% to 90% of renal malignancies. In recent years, an increasing number of studies have focused on the interaction between m6A modifications and circRNAs in RCC, which jointly regulate tumor progression and drug resistance. Researchers investigated circMET, derived from the MET gene in Xp11.2 translocation/NONO-TFE3 fusion renal cell carcinoma (NONO-TFE3 tRCC) (86). YTHDC1 promoted the cytoplasmic translocation of circMET through an N6-methyladenosine (m6A)-dependent mechanism, thereby enhancing CDKN2A mRNA decay and promoting the proliferation of NONO-TFE3 tRCC (86). In addition, the regulatory role of m6A modification of circPPAP2B in the proliferative and metastatic capacity of ccRCC cells. CircPPAP2B interacts with HNRNPC in an m6A-dependent manner, promoting HNRNPC nuclear translocation and facilitating ccRCC proliferation and metastasis (87). CircPOLR2A plays an important role in the proliferation and metastasis of clear-cell renal cell carcinoma (cRCC), with strong expression observed in metastatic cRCC tissues. In cRCC tissues, circPOLR2A regulates UBE3C-mediated ubiquitination and degradation of PEBP1 protein, further activating the ERK pathway during cRCC progression and metastasis. The m6A reader YTHDF2 regulates circPOLR2A expression in cRCC (88). Thus, circPOLR2A may serve as a potential target for the diagnosis and treatment of cRCC. Taken together, the complex interactions between m6A modifications and circRNAs in RCC onset, progression and drug resistance provide potential targets for developing new therapeutic strategies.

### 5.10 Hepatocellular carcinoma

Hepatocellular carcinoma (HCC) is the most common primary malignant liver tumor and is associated with high lethality. Genetic and epigenetic aberrations are frequently observed in HCC (104). m6A modifications and circRNAs act synergistically to promote cell proliferation in HCC cells. Specifically, m6A-modified circMDK activates the PI3K/AKT/mTOR signaling pathway via the miR-346/874-3p-ATG16L1 axis, thereby promoting cell proliferation (89). The complex interplay between m6A modification and circRNAs plays a critical role in HCC development treatment, and further investigation of their relationship may facilitate the identification of novel therapeutic strategies and biomarkers.

### 5.11 Esophageal squamous carcinoma

Esophageal squamous cell carcinoma (ESCC) is a malignant tumor originating from the epithelial squamous cells and is associated with high morbidity and mortality. It has been demonstrated that circCREBBP is closely linked to m6A modification and radiosensitivity in ESCC. CircCREBBP, modified by m6A, interacts with IGF2BP3 to reduce MYC mRNA stability, thereby enhancing ESCC radiosensitivity (90). Additionally, METTL3-mediated m6A modification upregulates the expression of circ0006168 in an IGF2BP2-dependent manner, promoting ESCC cell proliferation, migration, invasion, cell cycle progression, and inhibiting apoptosis (91). In conclusion, circRNAs modified by m6A may serve as potential therapeutic targets for ESCC.

### 5.12 Gastric cancer

Gastric cancer (GC) is the fifth most common cancer and the third leading cause of cancer-related deaths globally (105). mETTL14-mediated m6A modification of circORC5 inhibits GC progression by regulating the miR-30c-2-3p/AKT1S1 axis (92). circPAK2, through YTHDC1-dependent m6A methylation, is exported from the nucleus to the cytoplasm and interacts with IGF2BPs to form a circPAK2/IGF2BPs/VEGFA complex, stabilizing VEGFA mRNA and thereby promoting GC angiogenesis and invasiveness (93). This process highlights the critical role of m6A modification and circRNAs in tumor development and provides potential therapeutic targets for GC treatment.

### 5.13 Laryngeal squamous cell carcinoma

Laryngeal squamous cell carcinoma (LSCC) is a common malignancy affecting the head and neck region, causing severe impairment of voice, breathing, and swallowing functions. The presence of circMMP9 plays a critical role in determining the poor prognosis of LSCC, and its knockdown effectively attenuates the proliferation and metastasis of LSCC cells. Additionally, IGF2BP2 functions as an m6A reader to regulate the stability of circMMP9 (94). Li et al. demonstrated that EIF4A3-induced upregulation of circCDK1 inhibits the m6A modification of CPPED1 in an IGF2BP2-dependent manner, thereby promoting the progression of LSCC (95). These findings suggest that m6A modification-mediated circRNAs may serve as novel diagnostic and prognostic markers or potential therapeutic targets for LSCC.

### 5.14 Cervical cancer

Cervical cancer (CC) is the most prevalent gynecologic malignancy. However, the prognosis of recurrent and metastatic CC remains unsatisfactory, highlighting the need to identify new therapeutic targets to enhance the anti-tumor efficacy in advanced CC. Researchers identified a circRNA, circCCDC134, which was upregulated in CC tissues through circRNA-Seq analysis. This circRNA was primarily stabilized by ALKBH5-mediated m6A modification in a YTHDF2-dependent manner, thereby enhancing tumor proliferation and metastasis (96). Additionally, circ0000069 maintained its stability via m6A modification and specifically acted as a sponge for miR-4426, promoting CC cell proliferation and migration (97). Conversely, ZC3H13-mediated m6A modification of circ0081723 promotes CC progression by regulating the AMPK/p53 signaling pathway (98). These findings suggest that targeting circRNA demethylation may represent a promising therapeutic strategy and provide a novel regulatory model for investigating the oncogenic mechanisms of m6A-modified circRNAs in CC.

## 6 Clinical prospects of m6A modification-mediated circRNAs in cancer

### 6.1 As a cancer diagnostic and prognostic marker

Many m6A-modified circRNAs exhibit expression levels in cancer tissues or body fluids that are distinct from normal tissues and can serve as potential diagnostic markers. circNSUN2 expression is up-regulated in tissues and sera of patients with CRC liver metastases and correlates with poor prognosis (73). In addition, m6A-modified circSTX6 was highly expressed in HCC and CC and could serve as a potential marker for the diagnosis of these cancers (56). As circRNA has a covalent closed-loop structure, it is more stable than linear RNA, less susceptible to degradation, and able to persist in body fluids such as blood, facilitating its use as a marker for cancer diagnosis (106). In the serum of patients with CRC liver metastases, the expression level of circNSUN2 can be used as a diagnostic indicator (73). The expression levels of some m6A-modified circRNAs are closely related to the clinicopathological features of cancer, such as tumor size, stage, grading, and lymph node metastasis, and can be used as indicators for prognostic assessment. For example, high expression of circSTX6 in hepatocellular carcinoma was correlated with the aggressive phenotype of the tumor and predicted a poorer prognosis (107).

### 6.2 Providing new targets for cancer therapy

m6A-modified circRNAs and the pathways they regulate provide new targets for cancer drug development. For example, the METTL3/circSTX6/SPI1 feedback loop plays an important role in cervical cancer (56), and drug development targeting this loop is expected to be a new direction for cervical cancer treatment. In addition, FTO, as the first m6A demethylase identified, has become a hotspot for the development of targeted anticancer drugs. A variety of FTO-targeted inhibitors have been developed, including MO-I-500, meclofenamic acid (MA), FB23, R-2HG, and rhodopsin, which significantly inhibit the proliferation of cancer cells by inhibiting the enzymatic activity of FTO (108).

In summary, the use of m6A-modified circRNAs as biomarkers allows for early screening and evaluation of drug efficacy. By detecting the changes in circRNA expression before and after treatment, the inhibitory effect of drugs on tumors can be assessed, providing a basis for drug development and clinical application.

### 6.3 Combination applications for cancer therapy

Removal of m6A modifications on certain circRNAs or disruption of their interactions with associated proteins results in enhanced sensitivity of tumor cells to chemotherapeutic drugs. For example, disruption of CENPA-m6A-cenRNA interactions results in abnormal chromosome segregation and genomic instability in cancer cells, inhibits cancer cell growth, and enhances their sensitivity to mitogen-associated drugs (109). m6A-modified circRNAs may be involved in regulating the tumor microenvironment, attenuating chemotherapy-induced side effects such as immunosuppression by modulating immune cell function or cytokine expression. side effects such as immunosuppression. Resistance of some cancer cells to radiotherapy is one of the important reasons for treatment failure. Studies have shown that m6A-modified circRNAs play a key role in the development of radiotherapy tolerance. circCUX1 mediates radioresistance by binding to the mRNA 3’-UTR of Caspase-1 (76). circRNF13 is a novel circRNA modified with N6-methyladenosine (m6A), which is capable of enhancing the expression of Caspase-1 by enhancing the expression of Caspase-1 (76). circRNF13 is a novel circRNA modified by N6-methyladenosine (m6A). RNA, which is able to enhance the radiation tolerance of CC cells by enhancing the stability of CXCL1 mRNA, which in turn enhances the radiation tolerance of cervical cancer (110).

m6A-modified circRNAs regulate the function of immune cells and enhance the body’s immune response to tumors. By regulating m6A-modified circRNAs, the maturation and antigen-presenting ability of dendritic cells can be affected, thereby enhancing T cell-mediated immune responses (111, 112). m6A-modified circRNAs can affect immune cell infiltration and cytokine secretion in the tumor microenvironment, thereby regulating the immune microenvironment and enhancing the efficacy of immunotherapy (78, 113).

The mechanism of m6A-modified circRNAs in cancer has not been fully clarified and further in-depth studies are needed. In addition, the specificity and safety of m6A-modified circRNAs as therapeutic targets still need to be verified. In terms of clinical application, the detection and monitoring techniques of m6A-modified circRNAs still need to be further optimized. With the in-depth study of the mechanism of m6A-modified circRNAs in cancer and the continuous progress of detection and monitoring technologies, m6A-modified circRNAs are expected to play a greater role in the diagnosis, prognosis and treatment of cancer. In the future, m6A-modified circRNAs are expected to be used in combination with chemotherapy, radiotherapy, immunotherapy and other therapeutic means to provide more effective treatment options for cancer patients.

## 7 Conclusions and perspectives

Although significant progress has been made in cancer treatment, satisfactory therapeutic outcomes have yet to be achieved due to issues such as drug resistance. Scientists have been actively exploring new cancer treatment targets, including circRNAs. CircRNAs are a class of ncRNAs with a closed loop structure, and their abnormal expression can regulate various activities, including apoptosis, proliferation, autophagy, and cell necrosis. Moreover, some abnormally expressed circRNAs can serve as biomarkers for diseases, particularly cancer (12). With advancements in circRNAs and m6A research, it has been revealed that m6A modification plays an extremely critical role in circRNA function. However, the investigation of m6A modification, circRNAs or m6A-modified circRNAs in cancer remains insufficiently comprehensive and in-depth. Further exploration of the relationship between m6A modification, circRNAs, and cancer may uncover new avenues for research and could become a novel hotspot in cancer studies.

In this review, by analyzing numerous scholarly articles on circRNA and cancer, we found that m6A modification is present in many circRNAs and participates in the regulation of its biogenesis, subcellular localization, and degradation through m6A-mediated mechanisms, potentially leading to abnormal expression and movement of circRNAs. Simultaneously, we also observed that M6A modification not only occurs in circRNAs but also in RNA-binding proteins (RBPs) capable of binding to circRNAs, which are closely associated with key tumor suppressor or anti-cancer factors in cancer. Research on m6A modification in cancer has progressed toward drug therapy applications (114). For instance, screening for ALKBH5 inhibitors revealed that imidazobenzoxazin-5-thione MV1035, a new sodium channel blocker, can inhibit ALKBH5, thereby reducing glioblastoma (GBM) invasion (115). Additionally, it was found that METTL3 deletion enhances the sensitivity of pancreatic cancer cells to anticancer drugs, such as gemcitabine, 5-fluorouracil, and DDP, while having minimal effects on cell morphology and proliferation (116). The discovery and application of these m6A targeted modulators may provide new and effective strategies for cancer treatment and overcoming drug resistance. Investigating the role m6A modification in circRNA-mediated cancer regulation represents an interesting topic worthy of further study, and more precise detection of m6A modification in circRNAs will depend on advancements in detection technologies.

CircRNAs regulated by m6A modifications have important research value as a potential therapeutic target, but their reliability in translational research still faces some challenges, especially in the presence of high mutation rates. m6A modification sites of circRNAs that are mutated may affect their binding to m6A recognition proteins, which in turn may alter their function. For example, a mutation in the m6A site of circZNF609 results in a reduction of its translation efficiency by approximately 50% (117). The uncertainty of such mutations may affect the reliability of circRNAs based on m6A modifications as therapeutic targets. Therefore, further studies are needed in the future to determine which m6A-modified circRNAs have critical roles in specific diseases and have relatively low mutation rates to improve target reliability.

In addition to m6A modifications, circRNAs (118), compared to other modifications that may be relatively limited in distribution and function. In addition, m6A modifications are highly conserved across species (118), which allows them to play key roles in a wide range of biological processes that other modifications may not possess. m6A modifications are uniquely advantageous and important in the regulation of circRNAs, and future studies will further reveal the potential for their application in biology and medicine. m6A modifications’ The dynamics of m6A modification is one of its important features, and further studies are needed to investigate the dynamics of m^6^A modification and its regulatory mechanism in different cellular states and environments in the future. Meanwhile, the m6A modification may be inter-regulated with other RNA modifications (e.g., m5C, m1A, etc.), and the interactions between these modifications and their biological significance need to be further investigated in the future.

The research on the effect of m6A modified circRNAs on cancer treatment resistance remains in a stage that requires substantial data support. Current detection methods for m6A modifications in circRNAs primarily include MeRIP-seq (119), methylation iCLIP (miCLIP), m6A label seq and DART seq (120–122). However, these methods have certain limitations and may be prone to false positives. Therefore, there is still a need to develop novel and more accurate detection techniques to identify m6A-modified circRNAs in cancer, thereby elucidating their roles in anti-cancer mechanisms and drug resistance.
